# Associations of Region and Lactation Stage with Odd-Chain Fatty Acid Profile in Triglycerides of Breast Milk in China

**DOI:** 10.3390/molecules27196324

**Published:** 2022-09-26

**Authors:** Yiren Zhang, Hui Zhang, Emad Karrar, Wei Wei, Wei Zeng, Qingzhe Jin, Xingguo Wang

**Affiliations:** 1 State Key Laboratory of Food Science and Technology, School of Food Science and Technology, National Engineering Research Center for Functional Food, International Joint Research Laboratory for Lipid Nutrition and Safety, Collaborative Innovation Center of Food Safety and Quality Control in Jiangsu Province, Jiangnan University, Wuxi 214122, China; 2 International Joint Laboratory on Food Safety, Jiangnan University, Wuxi 214122, China; 3 School of Basic Medicine, Gannan Medical University, Ganzhou 341000, China

**Keywords:** breast milk, odd-chain fatty acids, pentadecylic acid, margaric acid

## Abstract

Odd-chain fatty acids (OCFAs), with potential value for growing infants, have been reported in breast milk. The association of location and lactation stage with the profile and content of OCFAs in breast milk was studied. We analyzed 1487 breast milk samples collected from 12 areas in China, and 102 infant formulas from different brands were purchased from the local supermarket. The content of sn-2 C15:0 significantly decreased from the colostrum to the mature stage, while that of C17:0 was not significantly increased by the lactation stage (*p* > 0.05). The content of C15:0 and C17:0 significantly decreased dramatically after the colostrum period, while the content of C13:0 was highest in the mature stage. The level of C15:0 and C17:0 in human milk from Gansu and Xinjiang was significantly higher than that from other areas. Similar trends were observed on the level of sn-2 C15:0 and C17:0, whereas the content of sn-2 C11:0 and C13:0 was significantly higher in breast milk from Shandong. Based on the PDS-LA analysis, the difference among infant formulas, each stage of human milk and human milk from different locations were different. Research is needed to determine if there are health benefits associated with OCFAs.

## 1. Introduction

Breast milk is considered the ideal food for neonates, since it provides complete nutrition for neonates’ immunological protection and development. Compared with other nutritional components in human milk, the fat not only supplies energy, but also essential fatty acids, including arachidonic acid (20:4 *n*−6) and docosahexaenoic acid (22:6 *n*−3). Therefore, the distribution and composition of fatty acids in human milk have been universally recognized as the gold standard for designing fatty acids in infant formulas. It is already known that more than 200 fatty acids have been identified in human milk, but these acids were mainly those with an even number of carbons between 4 and 22 [1,2]. Although the composition of major fatty acids (>1%) in commercial formulas were similar to human breast milk, some micro-fatty acids have been well designed. 

Odd-chain fatty acids (OCFAs) have been detected in brain tissue, adipose, buccal, liver, red blood cells and human plasma [3], and are also reported as compounds with anti-inflammatory, anticarcinogenic, antioxidant, antibiotic, and non-cytotoxic immunosuppressive activity, which has been reviewed by Rezanka and Sigler (2009) in detail [4]. Imamura et al. (2018) have shown that pentadecanoic (C15:0) and heptadecanoic (C17:0) acids are the main OCFAs in serum and human plasma [5]. OCFAs could be produced endogenously or originate from some food sources, such as cow milk, sheep milk and human milk, and it has also been proposed that dairy products might be the major food source of OCFAs [6]. It has been shown that the levels of OCFAs in human breast milk vary by population or country. Fox example, the contents of C15:0 and C17:0 in breast milk from the Barcelona metropolitan area in Spain were between 0.19% and 0.26%, between 0.25% and 0.30%, respectively, while human milk from five regions in China had the different levels of C15:0 (0.11–0.17%) and C17:0 (0.21–0.25%) [7]. However, these studies were only analyzed with smaller sample sizes and during late-stage lactation. 

The physiological function of human milk not only depends on the composition of fatty acids, but also the distribution of fatty acids in triglycerides. The data reveal that 70% of palmitic acid is distributed at the sn-2 position in triglycerides, while unsaturated fatty acids esterify in sn-1/3 positions in human milk triglycerides [8,9]. The absorption of calcium and fat, as well as the growth of beneficial bacteria, such as Lactobacillaceae and Bifidobacteriaceae, could be significantly improved by the intake of human milk with oleic–palmitic–oleic acid [10]. For OCFAs, breast milk is the leading provider to infants before they can be fed animal milks or primary foods containing OCFAs. Although the total profile and content of OCFAs in human breast milk have been reported, the sn-2 position-specific distribution of OCFAs has not been well studied. Therefore, the stereochemical distribution of OCFAs in human milk triglycerides should be studied. 

Therefore, the purpose of this study was to determine the association of location and lactation stage with the OCFA profile and the sn-2 position distribution in triglycerides of breast milk in China.

## 2. Results

### 2.1. Associationof Lactation Stage with the Content of OCFAs

**Figure 1:**
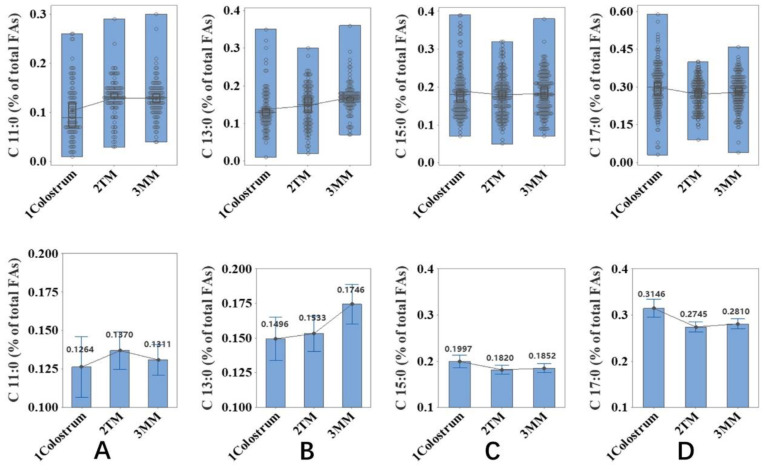
Association between lactation stage and the odd-chain fatty acids ((**A**): C11:0, (**B**): C13:0, (**C**): C15:0, (**D**): C17:0) in human milk. Undecylic acid = C11:0; Tridecylic acid = C13:0; Pentadecylic acid = C15:0; Margaric acid = C17:0; TM = Transitional milk; MM = Mature milk.

A total of 487 milk samples (group 1) from Wuxi with the known information on the milk stage, including colostrum (*n* = 161), transition milk (*n* = 163), and mature milk (*n* = 163) were collected to investigate the percentage amount of the concerned OCFAs. The OCFAs found in these milk samples are four saturated fatty acids: undecylic acid (C11:0), tridecylic acid (C13:0), pentadecylic acid (C15:0) and margaric acid (C17:0), and two mono-unsaturated fatty acids, namely C15:1 *n*−7 and C17:1 *n*−7, were also found. One-way ANOVA was used to determine the changes of milk OCFAs brought about by longer breastfeeding time in group 1. As seen in Figure 1, lactation stage had a significant influence on the content of C11:0, C13:0, C15:0 and C17:0 (*p* ≤ 0.05). The content of C13:0 was the highest in the mature stage. Natural milk had the lowest content of C11:0, while C15:0 and C17:0 had the highest content in the colostrum stage. The level of C13:0 significantly increased by 15% during the conversion from colostrum to transition milk, but the value of C15:0 and C17:0 decreased by around 10% from colostrum stage to transition stage. 

### 2.2. Association of Lactation Stage with the Content of sn-2 OCFAs

**Figure 2:**
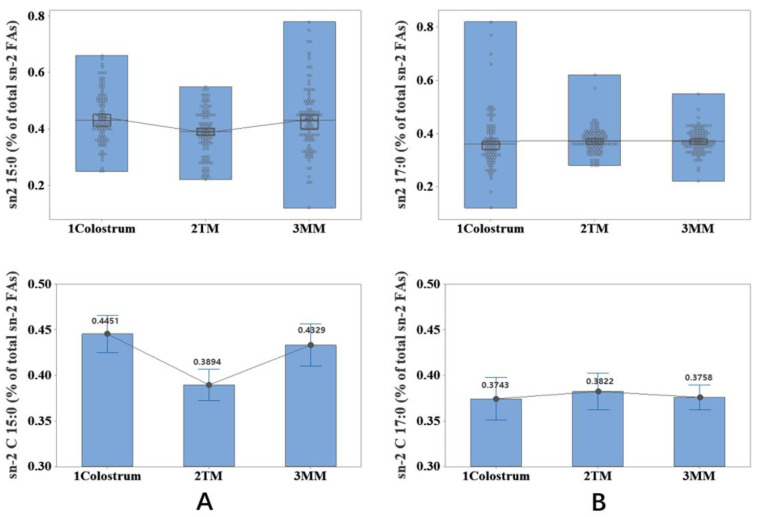
Association between lactation stage and the odd-chain fatty acids at sn-2 position ((**A**): C15:0, (**B**): C17:0) in human milk. Undecylic acid = C11:0; Tridecylic acid = C13:0; Pentadecylic acid = C15:0; Margaric acid = C17:0; TM = Transitional milk; MM = Mature milk.

The ANOVA test against the sn-2 OCFAs, namely sn-2 C11:0, sn-2 C13:0, sn-2 C15:0 and sn-2 C17:0, gave a rather inconsistent result. As shown in Figure 2, sn-2 C11:0 and sn-2 C13:0 were not determined in three stages. C15:0 at the sn-2 position varied significantly (*p* < 0.005) between different milk stages, with colostrum and transition being the highest and lowest, respectively. In comparison, the sn-2 C17:0 content was not significantly influenced by lactation stage (*p* > 0.05), and sn-2 C17:0 was nearly the same over the 90 days of milk stages, at a level of 0.38. 

### 2.3. Associationof Milk Origins with the Content of OCFAs

**Figure 3:**
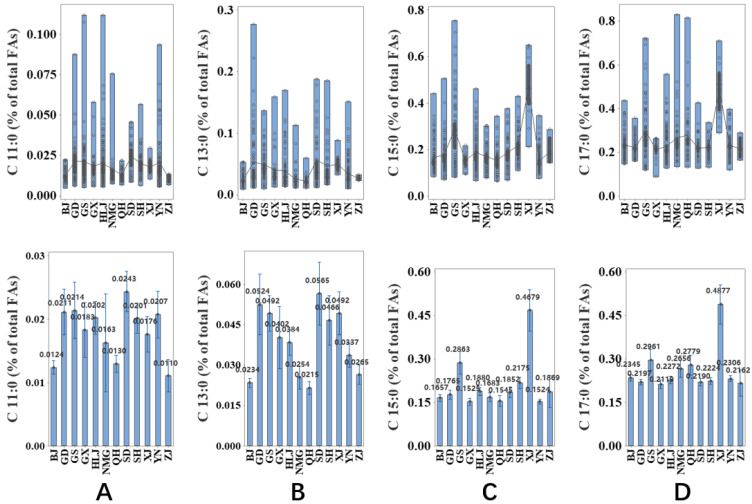
Association between location and the odd-chain fatty acids ((**A**):C11:0, (**B**): C13:0, (**C**): C15:0, (**D**): C17:0) in human milk. Undecylic acid = C11:0; Tridecylic acid = C13:0; Pentadecylic acid = C15:0; Margaric acid = C17:0; BJ = Beijing; GS = Gansu; GD = Guangdong; GX = Guangxi; HLG = Heilongjiang; NMG = Neimenggu; QH = Qinghai; SD = Shangdong; SH = Shanghai; XJ = Xinjiang; YN = Yunnan; ZJ = Zhejiang.

A total number of 778 human milk samples were collected from 12 provinces, including Yunnan (109), Shanghai (60), Shandong (63), Qinghai (49), Neimenggu (58), Heilongjiang (127), Guangxi (30), Guangdong (80), Gansu (72), Zhejiang (6), Xinjiang (15) and Beijing (109), and the association of location with the content of OCFAs is shown in Figure 3. The content of four different OCFAs was significantly influenced by location (*p* ≤ 0.05). With regard to C11:0 (Figure 3A), the highest value was found in samples from Gansu, but the human milk from Qinghai had the lowest content. As shown in Figure 3B, the level of C13:0 in samples from Bejing, Qinghai and Neimenggu was extremely lower than that of samples from other provinces. For C15:0, human milk from Xinjiang had the highest content, followed by that from Gansu, and a similar content was found in samples from the rest of the provinces (Figure 3C). Regarding C17:0, shown in Figure 3D, the highest content was also reported in human milk from Xinjiang, followed by that from Gansu, while samples from Qinghai and Neimenggu had a similar content level. 

### 2.4. Associationof Milk Origins with the Content of sn-2 OCFAs

**Figure 4:**
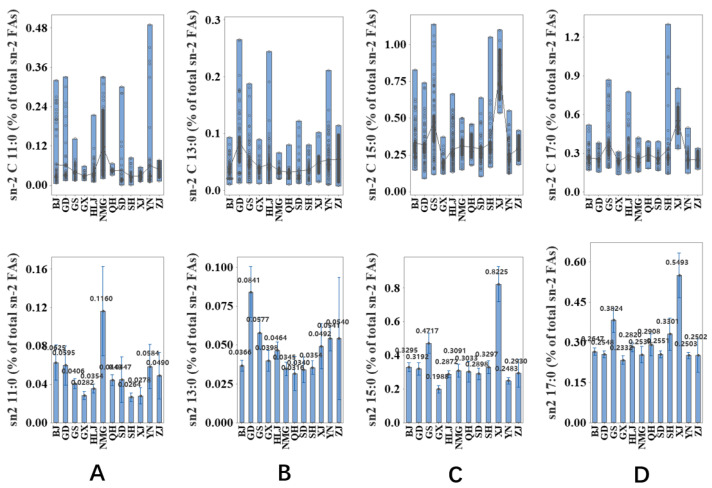
Association between location and the odd-chain fatty acids at sn-2 position ((**A**): sn-2 C11:0, (**B**): sn-2 C13:0, (**C**): sn-2 C15:0, (**D**): sn-2 C17:0) in human milk. Undecylic acid = C11:0; Tridecylic acid = C13:0; Pentadecylic acid = C15:0; Margaric acid = C17:0;BJ = Beijing; GS = Gansu; GD = Guangdong; GX = Guangxi; HLG = Heilongjiang; NMG = Neimenggu; QH = Qinghai; SD = Shangdong; SH = Shanghai; XJ = Xinjiang; YN = Yunnan; ZJ = Zhejiang.

The association of location with the content of sn-2 OCFAs in human milk from 12 provinces is shown in Figure 4; the content of sn-2 OCFAs was significantly influenced by location (*p* ≤ 0.05). The content of sn-2 C11 in human milk from Neimenggu was higher than that from other provinces, but the lowest value was found in samples from Shanghai (Figure 4A). As shown in Figure 4B, human milk from Guangdong had the highest content of sn-2 C13:0, while the content of sn-2 C13:0 in samples from Qinghai was the lowest. In terms of sn-2 C15:0 and sn-2 C17:0, the highest content was found in samples obtained from Xinjiang, followed by samples from Gansu, while the lowest level was determined in Guangxi (Figure 4C,D). 

### 2.5. Analysis of the OCFAs in Infant Formula

**Figure 5:**
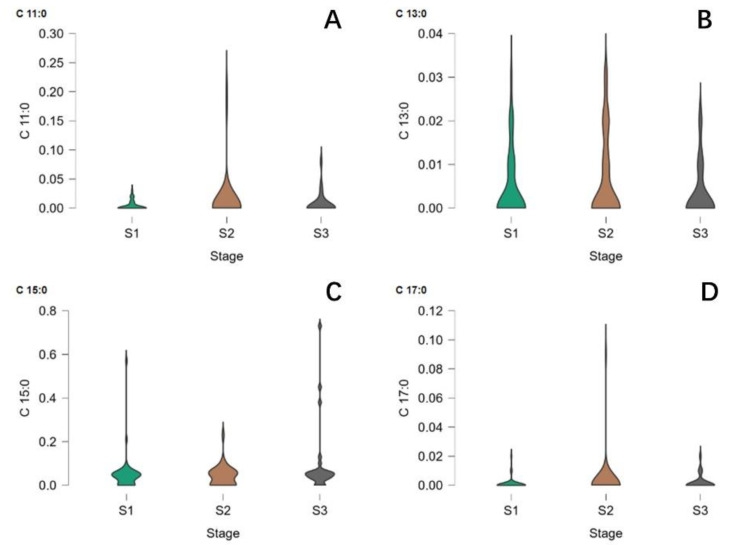
Comparison of OCFA contents in different infant formulas. Violin plot of (**A**): C11:0, (**B**): C13:0, (**C**): C15:0, (**D**): C17:0 contents in infant formulas. S1—Stage 1 infant formula; S2—Stage 2 infant formula; S3—Stage 3 infant formula.

A total of 102 infant formula samples from 24 brands were analyzed, as presented in Figure 5. In total, 41 were Stage 1 formulas, whereas 34 and 26 were Stage 2 and Stage 3, respectively. The content of C11:0, C13:0, C15:0 and C17:0 was significantly influenced by infant formula brand (*p* ≤ 0.05), and it can be seen that the average content of OCFAs was highest in Stage 3 formula, followed by Stage 2 and 1. Formulas from different brands also vary, the OCFA level of formulas from 10 brands were untestable, regardless of the formula stage.

### 2.6. PDS-LA Analysis of the OCFA Profile

**Figure 6:**
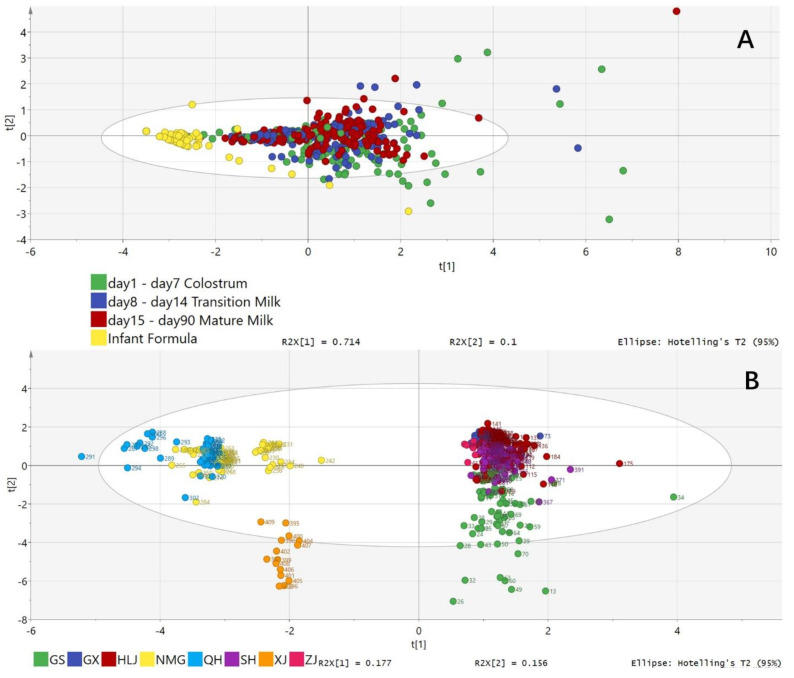
PDS-LA analysis of (**A**): the difference between infant formulas and different human milks (Green: Colostrum, Blue: Transition milk, Red: Mature milk, Yellow: infant formula) and (**B**): PDS-LA analysis of the difference of OCFA contents between human milks from different locations. GS = Gansu; GX = Guangxi; HLG = Heilongjiang; NMG = Neimenggu; QH = Qinghai; SH = Shanghai; XJ = Xinjiang; ZJ = Zhejiang.

PDS-LA analysis was used to compare the total OCFA contents in infant formulas with those in different stages of human milk in group 1; results are shown in Figure 6A,B. It was found that the OCFAs in infant formulas were different from those of each stage of human milk. The difference between infant formulas and human milk from different locations was distinctive (Figure 6B). Based on the PDS-LA analysis of the OCFAs, twelve provinces could be divided into two regions. Gansu, Guangxi, Heilongjiang, Shanghai and Zhejiang were similar, while Neimenggu, Qinghai and Xingjiang were alike. The OCFAs in human milk from the two regions were significantly distinctive.

## 3. Discussion 

As stated in the introduction, OCFAs have been proven to have several health benefits to general human beings. Pentadecylic acid (C15:0) and margaric acid (C17:0) are the most abundantly researched OCFAs. The demonstration of such an association mainly relates to the metabolic pathways of OCFAs and previous statistical causal relationships [11]. The possible final products of OCFA biosynthesis are C23:0 and C25:0, which are known to be considerably involved in gangliosides. This glycosphingolipid is indispensable at a high level in the human brain [12]. 

Other studies also reported that the OCFA content of people with chronic diseases, such as cerebrovascular disease and diabetes, was lower than that of healthy people. 

For babies, infant formulas or breast milk are the main source of OCFAs. In terms of human milk, the level of these two main OCFAs is a total of 0.38% to 0.48% fatty acids. Such a level is set between the level of Eicosapentaenoic acid (EPA) and Docosahexaenoic acid (DHA) in human milk [13]. In 487 samples, the level of both OCFAs showed more nonnegligible alteration during colostrum–transition milk transformation than transition–mature milk transformation (Figure 1). The first proof of the fundamental change of OCFAs in different infant milk is the human infant intestinal microbiota. A DNA sequence of infant feces revealed that the microbiota varies hugely during the growth of a human infant. Specifically, the microbiota in the meconium consisted of >99% firmicutes, which represented the gut microbiota of the unborn child. Subsequently, the detection rate of proteobacteria in the feces of fetuses 6 days after birth increased to nearly 10% and replaced firmicutes.. A further 75% of fungi were found in the feces of fetuses on day 85 of life. [14]. These three patterns share the exact thresholds of the colostrum and transition/mature milk stages, coincidently. Before being delivered, the infants obtain nutrition directly from their mother’s blood through the umbilical cord, and then they receive nutrition via eating and metabolism. The change of gut microbiota colony from meconium to fecal samples could be influenced by the food of the infant, namely human milk or formula. As a result, the comparatively higher level of OCFAs was observed in the colostrum milk stage, indicating the demand of mothers to give sufficient fuel for newborn infants to cultivate their multi-component gut microbiota. During the transition/mature milk stage, this demand fades, as infants are more able to intake complicated food as supplies for their growing gut microbiota, leading to the decrease in OCFAs in human milk during these stages.

The variance of OCFAs might also be related to brain development. Glycosphingolipids are prevalent in the nervous system. During neurodevelopment, especially in the early stages, dramatic, constant alterations in ganglioside expression are seen [15]. The OCFAs C15:0 and 17:0 could be lengthened to very-long-chain FAs (VLCFAs), such as tricosanoic acid (23:0) and pentacosanoic acid (25:0) [7]. This is also consistent with the pattern of OCFA changes in Figure 1.

The OCFA composition at the sn-2 position showed a rather disparate pattern regarding total composition. It can be seen in Figure 2 that the level of sn-2 C17:0 was constant within the three milk stages (*p* = 0.83), but the sn-2 C15:0 level dropped during the transition milk period and rose afterward. (*p* < 0.001). Yet, some anomalies were seen in all three periods with several-times-higher values. Compared with total C15:0, sn-2 C15:0 was relatively higher in mature milk, whereas such a pattern happened in transition milk when C17:0 was compared with sn-2 C17:0. The point showed that fatty acids at the sn-2 position are more conducive to absorption and might contribute to the sn-2 OCFA inconsistency [16]. Hence, C17:0 was likely more valuable in the transition milk period.

Regarding the region-wise analysis of OCFAs, a significant difference can be seen in the samples from Xinjiang and Gansu, as shown in Figure 3 and Figure 4. In contrast, the results from other regions appear to be similar, regardless of the vast geographical span. Several hypotheses can be proposed for this phenomenon. A possible factor is the consumption of yak milk products. It was found that the level of C15:0 in full-lactoseyak milk in Qinghai was 3.84%, and that of C17:0 was 2.51% [17]. Gansu and Xinjiang are the main yak-producing regions. The density of population of these two provinces is the second lowest and the lowest among all twelve attractive regions (62 people/sq.km and 15 people/sq.km, respectively), and the altitude of the provincial capital is also relatively higher (1520 m and 917.9 m, respectively), which restricts the cross-regional circulation of yak milk. Therefore, a relatively high proportion of yak milk consumption in dairy product consumption is predictable in these two provinces, and the levels of C15:0 and C17:0 in yak milk were either found to be undetectable [18] or minor (1.32 and 0.58, respectively) [19], leading to a similar pattern of OCFAs in human milk [20]. Unlike the inconsistent practices of sn-2 OCFAs and total OCFAs in the first group, such patterns in the region-wise groups are obviously accordant. As presented in Figure 4, the sn-2 C15:0 and sn-2 C17:0 content was the highest in Xinjiang, followed by Gansu, while the levels in other regions were not significantly different. The possible explanation is that the regional difference in body might not influence the OCFA geometry in TAGs as much as the milk stage [21].

The total OCFA level of human milk from several cities was relatively close. However, as stated in the results, it is observed that the cities were divided into two clear clusters (Figure 6). The pattern of this division followed the rule of the three steps of China’s terrain: the second geographical step, including Xinjiang, Qinghai and Neimenggu; and tThe third geographical step, which includes the rest of the provinces [22]. These findings further support the hypothesis that dietary patterns remarkably influence the level of OCFAs in breast milk, as such patterns are generally believed to be related to geographic zoning. It is known that a more significant proportion of citizens from cities that belong to the second step are Muslims, which implies a Halal diet apart from the traditional Han diet, such as the exclusive preference for beef instead of pork [23]. 

It was also found that the level of OCFAs detected in human milk from the two groups had some inconsistencies. According to a previous report, vegans, vegetarians and omnivores have similar OCFA concentrations in their red blood cells, and the content of C17:0 was higher than that of C15:0 in human plasma [11,24]. The average level of C15:0 varied mainly between the first and the second group (38.46%), while that of C17:0 was mild (16.00%). Therefore, the proportion of vegetable intake versus OCFA-containing food intake, such as ruminant fats, could affect the level of OCFAs in secreted milk. Nevertheless, such an observation could also imply that the alteration of OCFA levels in breast milk does not happen simultaneously in all human beings.

It can be clearly seen in Figure 5 and Figure 6 that the general OCFA composition of infant formula samples was rather low. When looking at the 41 Stage 1 infant formula samples, the C15:0 level was below 0.05% in 82% of them. Similarly, the level of C17:0 in 39% of the samples was below 0.05%. Such a pattern was similar in the Stage 2 infant formulas. Both OCFAs were below 0.05% in 60% of samples. These two FAs can be found at over 0.05% in over half of the samples belonging to Stage 3 formulas. It was universally accepted that the Stage 1 infant formula was guided to feed infants aged 0–6 months, whereas the Stage 2 formula was designed for babies between 6 and 12 months of age. Babies were given the Stage 3 formula over one year old [25]. When compared to breast milk, Stage 1 infant formula should be considered a replacement for all types of milk, including colostrum milk, transition milk, and mature milk. It was believed that the Stage 1 procedure aimed to imitate breast milk’s nutrition value as much as possible. Yet, the content of OCFAs was dissatisfactory, as the level of these FAs in the breast was proved. The lack of OCFAs in Stage 1 and Stage 2 infant formula and the presence of OCFAs in Stage 3 infant formula, may be due mainly to the difference in OCFA content in the raw materials used in the production of the formula [26]. To meet a certain level of other valuable fatty acids, such as DHA and EPA, certain oils containing the required nutrients were used. For example, to meet the criterion of palmitic acid (PA) in infant formula, palm oil has been chosen as a cost-effective raw material. Therefore, it could be indicated that the OCFA was not currently considered a special requirement in infant formula, especially in the Stage 1 and 2 formulas. The observation of the existence of OCFAs in Stage 3 infant formula might indicate that many Stage 3 formulas used cow’s milk as a raw material, which, unlike vegetable oil, contains a decent amount of OCFAs; the possible reason might be that infants over 1 year-old are already able to eat food. In summary, most suitable infant formulas cannot provide OCFAs to infants younger than one year. 

Although the levels of C15:0 and C17:0 vary over time in breast milk, PDS-LA analysis of breast milk OCFAs was not effective in distinguishing between the different stages of breast milk, as presented in Figure 6. However, when comparing infant formula with breast milk at various locations, there were significant differences between OCFAs and infant formula at each stage of breast milk. This difference could be mainly attributed to the raw materials used, as discussed before. Nevertheless, as shown in Figure 6A, colostrum milk samples were relatively less united, followed by transition milk compared with infant formula samples. Mature milk samples were highly consistent with no overlaps in PLA-DS compared with infant formula samples.

## 4. Materials and Methods 

### 4.1. Data Collection and Statements

#### 4.1.1. Infant Formula Collection

A total of 24 common brands of infant formula were purchased from local supermarkets, and some brands with sub-brands of infant formula or different stages of infant formula were also included. In total, 102 different infant formulas were used. The tags and outer packages of those formulas were disposed of for unbiased results. However, the stages of those infant formulas were recorded. In total, 41 formulas were in the Stage 1, and 34 and 26 were Stage 2 and Stage 3, respectively. 

#### 4.1.2. Human Milk Sample Collection

All breast milk samples from interested donors were used in this investigation. We provided detailed information to participants about the study, and they were required to sign a consent form. For the milk samples from Wuxi, The Ethics Committees of institutions that are involved (Jiangnan University Medical Research Board, Hospital of Wuxi Maternity and Child Health Care) accepted the study procedure (WXM201560) [9]. The volunteers lived in Wuxi, China, and were free of health conditions and had healthy children, according to their medical histories and physical examinations. The women’s gestation periods varied from 27 to 39 weeks, and their average age was 27.86. Parity was 1.2 on average. Breast milk was obtained 1–7 days after birth (colostrum), 8–14 days after birth (transitional milk), and 15–90 days after birth (mature milk). Before the sample collection, the participants were given oral and written instructions for standardized sample collection. At 10 a.m., a breast massage was used to extract a portion of the entire breast milk. The very first drop of milk was discarded. The samples were kept frozen at −20 °C until they arrived at the lab, and then at −80 °C until they were tested. Jiangnan University received all of the models for storage, processing, and lipid analysis [9]. For other cities or provinces, the milk samples were kindly collected by local hospitals and delivered to Jiangnan University in Wuxi under cold-chain transportation at −20 °C until they arrived at the lab, and then at −80 °C until they were tested. The hospitals involved are as follows: Peking Union Medical College Hospital in Beijing; The first people’s hospital of Lanzhou city in Gansu; Guangdong Provincial People’s Hospital in Guangdong; Heilongjiang Provincial Hospital in Heilongjiang; HUHHOT First Hospital in Neimenggu; Qinghai Provincial People’s Hospital in Qinghai; The Affiliated Hospital of Qingdao University in Shandong; Huashan Hospital in Shanghai; The First Affiliated Hospital of Xinjiang Medical University in Xinjiang; Yunnan First People’s Hospital in Yunnan; and Zhuji People’s Hospital in Zhejiang. All hospitals that donated milk samples were given the same ethical study procedure (WXM201560) approved by Hospital of Wuxi Maternity and Child Health Care. The detailed method of milk collection varied slightly due to custom differences in hospitals from different regions. However, the mothers who willingly donated their milk were all healthy and aged between 24 and 32.

### 4.2. Method for Data Collection

#### 4.2.1. Total Lipid Extraction from Human Milk and Infant Formula Milk

The modified Mojonnier technique for extracting total lipids from milk was used (AOAC, 2000; method 995.19). We mixed 1 mL of ammonia water into 5 mL of milk completely and incubated it at 65 °C in a water bath for 20 min. Once the liquid had cooled, we added 5 mL of pure ethanol, 12.5 mL of ether, and 12.5 mL of ligarine to extract the lipids. After at least one hour for the samples to stand, the clear supernatants were collected. The lipids were again extracted, and the two fractions were combined. Then we used nitrogen blowing to eliminate any remaining solvent [25].

#### 4.2.2. Preparation of Fatty Acid Methyl Esters (FAMEs) for Chromatographic Analysis

To make the fatty acid methyl esters for chromatographic analysis, 10 mg of milk lipids were suspended in 700 L of *n*-hexane and 125 L of potassium hydroxide methanol (2 M). We mixed in 25 liters of sodium methoxide for 5 min before sodium sulfate was added and vigorously mixed it for 2 min. We then collected the supernatant and put it through a 0.22 m filter after standing. The produced FAME was examined using gas chromatography (GC).

#### 4.2.3. Preparation of 2-Monoacylglycerol and its Methyl Esters

Milk lipids were hydrolyzed to 2-monoacylglycerol using the technique reported by Sahin et al. (2-MAG). We added pancreatic lipase (porcine pancreatic lipase, 30 mg) to a test tube which contained 30 mg of fat, followed by the addition of tris buffer (pH 8.0, 7 mL), bile salts (0.05 percent, 1.75 mL), and calcium chloride (2.2 percent, 0.7 mL). The mixture was incubated for 3 min in a water bath at 37 °C with stirring. It was vortexed for 30 s before being incubated at 37 °C for 3 min. It was vortexed once again and incubated for another 2 min. After cooling, we added 2 mL diethyl ether and centrifuged for 3 min at 2500× *g* rpm. We moved the supernatant to a separate tube and used nitrogen gas to evaporate the diethyl ether to a volume of 500 L. Using the developing solvents hexane/diethyl ether/acetic acid (50:50:1, *v*/*v*/*v*), the hydrolytic product on a silica gel G TLC plate was separated. We used diethyl ether twice to remove the band corresponding to 2-MAG (1 mL). Nitrogen gas was used to remove the solvent, and then we methylated the residue and examined through GC.

#### 4.2.4. GC Analysis

The GC was an Agilent 7820A with a TRACE TR-FAME capillary column and a hydrogen flame ionization detector (60 m 0.25 mm 0.25 m, Thermo Fisher Scientific, Waltham, MA, USA). Both the injector and the detector were adjusted at 250 °C. At 1.2 mL/min, they employed a 1:100 split ratio nitrogen carrier gas. The oven temperature was maintained at 60 °C for 3 min before increasing to 175 °C by 5 °C/min for 15 min, then to 220 °C by 2 °C/min for 10 min. By comparing the peak retention periods of the samples to a series of FAME standards, the FAMEs were found. The FA content was calculated as a percentage of weight (*w*/*w*) of total FAs with a carbon atom chain length of 422.

The percentage of FAs in TAGs was estimated using the formula relative percentage = (M/T 3) × 100, whereas M represents the percentage of FAs in TAGs and T represents the percentage of FAs at the sn-2 position. FA concentrations were expressed as a weight percentage (*w*/*w*) of total FAs found with a 422 carbon atom chain length.

### 4.3. Statistical Analysis Methods

We used JASP (version 0.15.0.0), SIMCA (version 14.1), and Minitab 20. The normality of the dataset was tested via the Shapiro−Wilk test. Q–Q plots were used for data sub-groups with a large sample size. Levene’s test was used to check for homogeneity of variance in the dataset. A t-test and analysis of variance were used in circumstances where the sub-groups of data matched the assumption criterion. To examine the differences in the total odd-chain FA and sn-2 FA profiles in various human types of milk and new-born formulae, a partial least squares discriminant analysis (PLS-DA) was used.

## 5. Conclusions

In this research, we investigated human milk from different milk stages and regions in China, and the OCFA components in assessable infant formulas were also determined. Four main OCFAs, including C11:0, C13:0, C15:0 and C17:0, were determined in samples, and their contents were significantly influenced by lactation stage. The level of C13:0 in mature milk was the highest, while that of C15:0 in colostrum samples was the highest.

For Sn-2 C15:0 and C17:0 in human milk, their content was lowest in the transition stage. When considering all OCFAs and Sn-2 OCFAs, human milk had visible differences when provinces and cities were divided by geographical steps. Dietary differences between regions, especially the specificity of the Halal diet, may be the main reason for this difference. The comparison of OCFA profiles demonstrated a distinct difference between infant formulas and human milk. Therefore, it is meant to intensify the importance of understanding the existence of OCFAs in human milk and guide the design of infant formulas at different stages.

## Data Availability

Not applicable.
